# How are you? Do people with inflammatory bowel disease experience response shift on this question?

**DOI:** 10.1186/s12955-015-0232-6

**Published:** 2015-05-06

**Authors:** Nancy E Mayo, Susan C Scott, Charles N Bernstein, Lisa M Lix

**Affiliations:** Division of Clinical Epidemiology, Ross Pavilion R4. 29, Royal Victoria Hospital Site, McGill University Health Center, Montreal, QC H3A 1A1 Canada; Division of Clinical Epidemiology, Ross Pavilion R4. 36 Royal Victoria Hospital Site, McGill University Health Center, Montreal, QC H3A 1A1 Canada; University of Manitoba Inflammatory Bowel Disease Clinical and Research Centre, University of Manitoba, 804F-715 McDermot Ave, Winnipeg, MB R3E 3P4 Canada; University of Manitoba, S113-750 Bannatyne Ave, Winnipeg, MB R3E 0W3 Canada

**Keywords:** Response shift, Measurement of change, Quality of life, Inflammatory bowel disease, Longitudinal

## Abstract

**Background:**

As individuals experience changes in their health, they may alter the way they evaluate health and quality of life. The purpose of this study is to estimate the extent to which individuals with IBD change their rating of health over time because of response shift (RS).

**Methods:**

This is a reanalysis of a population-based longitudinal study of IBD in Manitoba, Canada (n = 388). RS was examined using trajectories of the difference between observed and predicted health. Logistic regression and dual trajectories were used to identify predictors of RS.

**Results:**

Disease activity, vitality, pain, somatization, and physical and social function explained 51% of the variation in general health over two years with no evidence of RS in 82% of the sample. Negative RS was found for 8%, who initially rated health better than predicted; positive RS was found for 6%. The positive RS group was younger and had better baseline scores on measures of general health, hostility, pain, mental health and social and role function; less pain and better social function scores at baseline were predictors of negative RS.

**Conclusions:**

In conclusion, the majority of people with IBD did not demonstrate a RS indicating that the health rating over time was stable in relation to that predicted by known time varying clinical variables. This adds to the evidence that the single question on self-rated health is useful for monitoring individuals over time.

## Introduction

Health-related quality of life (HRQL) is a broad concept that encompasses multiple domains, including physical, psychological, social, and emotional health, as well as general or global perceptions of health [1,2]. Longitudinal studies about HRQL and other patient-reported outcomes rest on the assumption that the respondent’s interpretation of a construct remains constant over time. Increasingly, HRQL measures are being used in clinical trials to evaluate the efficacy of new treatments or interventions, population-based surveys to describe population health, and chronic disease cohort studies to understand the impact of health challenges and treatment over the disease course. In longitudinal studies, a question of primary interest is: “Is there a significant improvement or deterioration in global HRQL and its domains?”

As individuals experience changes in their health, they may change the way that they evaluate their health and quality of life. Response shift (RS) is defined as a change in an individual’s internal standards (recalibration), values (reprioritization), or conceptualizations of health (reconceptualization) [3]. It is theorized that RS occurs when individuals experience a significant health event (i.e., catalyst), such as a stroke, cancer treatment, or chronic disease diagnosis [4]. When RS is present in HRQL, conventional statistical analyses may not reveal any evidence of an observed change even when a true change exists. Moreover, RS may not affect all HRQL domains or all samples, sub-samples, or individuals equally, which can complicate longitudinal data analysis.

Methods to detect RS include: (a) ***Design-based methods*** that collect supplementary data to estimate the magnitude of RS, (b) ***Interview or focus group methods*** that collect qualitative information about individuals’ experiences of RS, (c) ***Preference-based methods*** that collect comparative data about changes in the rank-order of domains, and (d) ***Model-based methods*** based on measurement error, multivariate, and longitudinal statistical models [5].

The most common design based method is the ‘then’ test [6]. Individuals evaluate HRQL at the baseline occasion and then again at the post-test (or follow-up) measurement occasion. At the post-test, individuals are also asked to re-evaluate their HRQL at the pretest (baseline) assessment occasion. The difference between the original pretest score and the retrospective pre-test score estimates the magnitude of RS. The ‘then’ test has several limitations: it must be built in at the design stage, it can only be used to estimate recalibration, it is sensitive to recall bias and social acceptability bias, and it is problematic to use when there are multiple measurement occasions [7,8]. The other design based methods have similar challenges with respect to data collection and respondent burden.

Model-based methods include: (a) structural equation models (SEM) [9-13]; (b) random-effects models, (c) relative important measures, and (d) classification models. Of these methods, random-effects models are more easily applicable in the case of multiple time points. We previously [14] described a subject-specific model-based method of testing for RS in data collected at multiple occasions. This is a useful method when the pattern of change over time may be influenced by the course of progression of a health condition. The method focuses on an analysis of the pattern of residuals (the difference between predicted and observed HRQL scores) over time, classifying people into discrete RS groups. This method has been used in an inception cohort of individuals with stroke [14] and in a large volunteer study of individuals with prevalent multiple sclerosis (MS) [15]. These two samples yielded, not surprisingly, different prevalences of response shift: 33% for stroke and 1% for MS. The high rates found in the stroke cohort are in keeping with the concept of RS resulting from a catalyst; incident stroke is a strong catalyst with a definitive onset. MS, which shares commonalities with other chronic diseases such as inflammatory bowel disease (IBD) and rheumatoid arthritis, develops over a longer time period and severity fluctuates. However, for the MS sample studied for RS, the timing of measurements was determined by the individual and not a study protocol. Thus, individuals may have chosen to report only when they were feeling well, and the picture of disease impact over time may have been minimized.

RS is an important consideration when evaluating changes in disease outcomes because it can result in an overestimating of problems or benefits in some individuals, and an underestimation in others. In a research context, these effects could cancel, yielding a conclusion of no change [16,17]. IBD is a chronic inflammatory disease with two distinct entities: Crohn’s disease and ulcerative colitis. However, regardless of the type of disease, over its course, individuals will experience changes in the intensity and severity of their symptoms. When there is increased inflammation, the disease is considered to be in an active stage and the individual will experience a flare-up of the condition, which may severely affect participation in work and social functions [18,19]. Symptoms include pain, fatigue and diarrhea; IBD is also associated with mastery, distress and lower psychological well-being [20,21]. When the degree of inflammation is low, the individual usually has mild symptoms or is symptom-free. Medical and surgical management has varying success, with up to half of individuals with IBD experiencing relapses every year. All of these aspects take a toll on health and quality of life of persons with IBD [20,22,23].

In order to accurately interpret IBD impact over time, from both the perspective of an individual and for group comparisons, it is important to have methods to identify RS. The area of IBD is understudied for this phenomenon.

The purpose of this study is to estimate the extent to which RS occurs among individuals with IBD, and to identify predictors of RS.

## Methods

### Data

Data for this analysis is from the Manitoba IBD Cohort Study, a prospective longitudinal cohort study that is investigating the determinants of disease outcomes. Initiated in 2002, the Cohort Study includes persons from a population-based registry, which was established in 1995 at the University of Manitoba. The formation of this cohort is described in previous publications [20,21]. The present study is a reanalysis of the data, which consists of 388 respondents, 18 years of age or older and diagnosed within seven years prior to enrollment (mean 4.3 standard deviation 2.1), with data collected by mail questionnaire at 5 measurement occasions between study entry and 24-months. During this period, 34 respondents (8.8%) dropped out.

### Measures

Respondents in the Manitoba IBD Cohort Study provided demographic information and information on a wide spectrum of health outcomes related to disease activity, physical and psychological symptoms, physical, role and social function, perceived health, and social support. Physical symptoms cover pain, fatigue and vitality, as well as those relating to the gastrointestinal system. Psychological symptoms include anxiety, mood, and somatic and systemic symptoms.

The outcome for this study is the single-item from the SF-36 General Health Perception (GHP) subscale: “In general would you say your health is”, with response options Excellent, Very Good, Good, Fair, Poor (EVGGFP). The other sub-scales of the SF-36 assess physical and social function, pain, vitality, mental health, and physical and emotional role impact, and were used as potential predictors of EVGGFP. All subscales are scored from 0 to 100, with higher values indicating better health. Some questions refer to the past four months, others, a typical day. The SF-36 has been widely validated as a measure of perceived health status [24,25]. Disease activity was assessed using the Manitoba IBD Index [26], a single-item index for symptom persistence based on the previous six-month period, shown to have good validity. IBD-specific predictors were selected from the Inflammatory Bowel Disease Questionnaire (IBDQ) [27]: gastrointestinal symptoms, systemic problems (fatigue, energy, feeling unwell, sleep, weight), emotional dysfunction, and social difficulties during the last two weeks. The IBDQ is a commonly used and extensively validated measure of HRQL in IBD [28].

Negative psychological functioning was assessed using the Cohen Perceived Stress Scale (CPSS), which asks about feelings and thoughts during the last month. It is a validated tool for measuring the role of stress in disease [29,30].

The Brief Symptom Inventory (BSI) measures depressive symptoms from the past seven days in nine dimensions: somatization, obsession-compulsion, interpersonal sensitivity, depression, anxiety, hostility, phobic anxiety, paranoid ideation, and psychoticism [31].

The Multidimensional Scale of Perceived Social Support (MSPSS) [32], with 12 items, assesses the degree of support from family and friends, presumably currently.

### Statistical analyses

**Figure 1:**
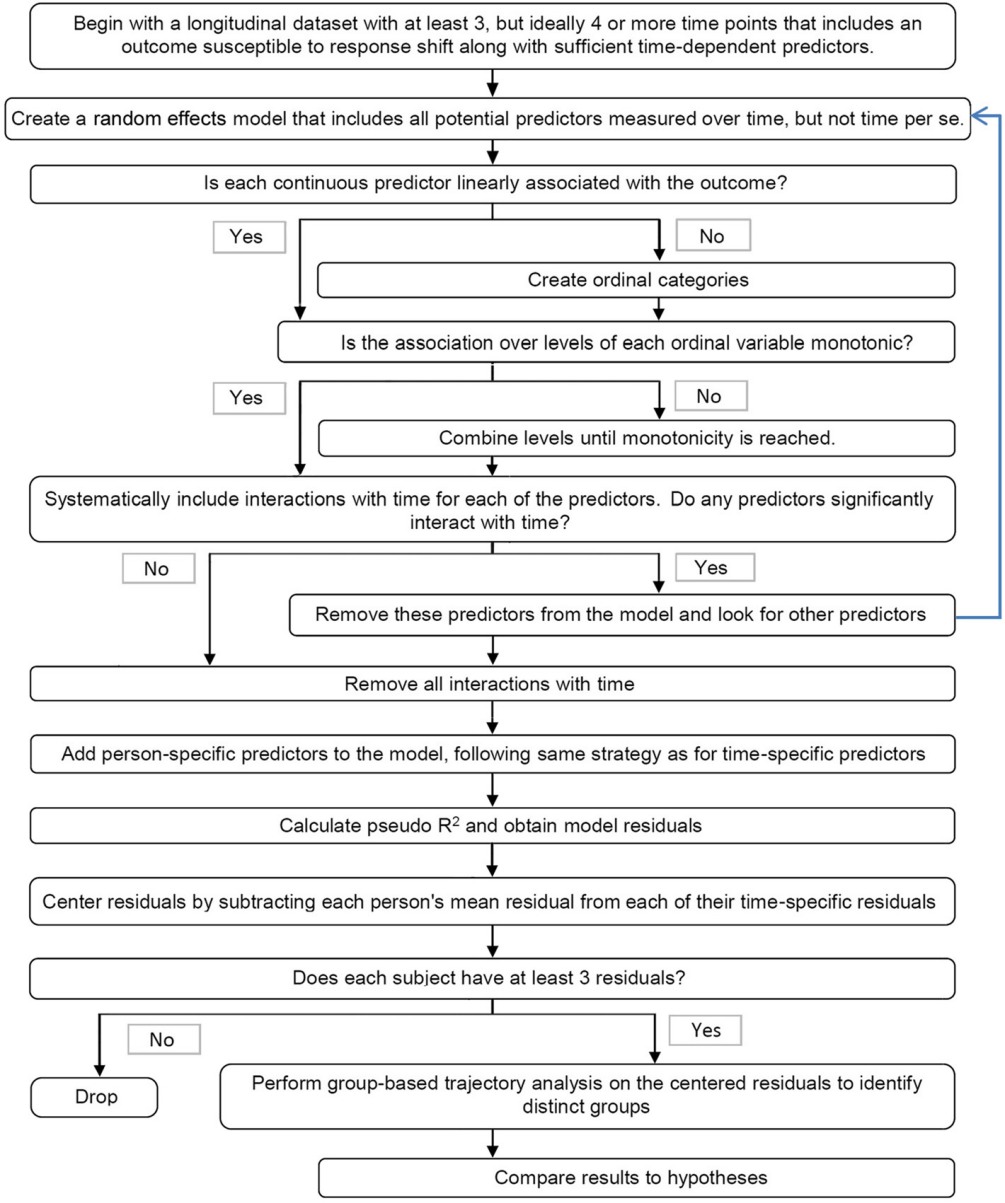
Flowchart of methods.

The statistical methods have been described previously [14,15] and Figure 1 outlines the steps. The method starts with the creation of the random-effects model using, as predictors of outcome (EVGFP), those variables measured over time: symptoms, functioning, perceived stress, and social support. All assumptions underlying linear models are tested and modifications made to ensure linearity and/or monotonicity. A key feature is that time is not in the model nor are any variables that interact with time, as time is considered part of the catalyst for RS. Assessments missing either the outcome or more than half of the predictor variables included in the final model were dropped. Remaining missing items were categorized with missing levels in the predictive model. Model fit was evaluated using Akaike Information Criterion (AIC) and Bayesian Information Criteria (BIC). A pseudo-R [2] was calculated to estimate the proportion of total variation in EVGGFP explained by the final model [33].

The detection of RS is based on the patterns of residuals over time, which are the differences between predicted EVGGFP and the observed value for each respondent at each time period. These residual values were centered by subtracting each respondent’s mean residual from each time-specific residual to focus on change over time rather than on each respondent’s deviation from expected. Persons with fewer than three residuals were excluded from further analysis.

Nagin’s group-based trajectory method [34] was used to identify respondents considered likely to have experienced a RS based on the patterns of residuals over time. Large fluctuations in a respondent’s observed and predicted health over time suggest that a RS has occurred, while a pattern of consistent residuals over time (though not necessarily close to predicted) suggests no response shift. GBTA is a form of latent class analysis for continuous outcomes based on the assumption that the population is a mixture of distinct groups defined by common change over time, while recognizing uncertainty in group membership. RS trajectories are qualitatively classified based on their shape and direction, reflecting the change in the centered residuals.

Posterior probabilities, representing a respondent’s likelihood of belonging to each of the trajectory groups, are provided for each respondent for each trajectory. They are used to calculate theoretical and assigned proportions. The theoretical proportion is an expected value calculated as the sum of posterior probabilities for that trajectory group over all respondents; the assigned proportion is calculated from each respondent’s highest posterior probability. Similarity between the theoretical and assigned probabilities is considered to indicate good model fit, as are respondents having a high posterior probability for a single group (measured using means of the group with the highest posterior probability). Other indicators of model fit are based on the AIC and BIC statistics, where values closer to zero indicate better fit. Two BIC statistics are produced, one based on the number of respondents, the other on the number of observations, with the correct but incalculable BIC lying between the two. Coherence with theory and all fit indices are considered in determining the number of trajectory groups and the number of parameters per trajectory (intercept, linear, quadratic, etc.).

Four response shift patterns were hypothesized based on other work [14]: none, positive, negative, or fluctuating. No response shift is likely when the centered residuals form a flat trajectory at 0: the difference between predicted and observed is consistent across time. Positive RS is deemed present when the magnitude of the residual increases over time in a direction of a more positive rating. This includes respondents who start with health ratings worse than predicted but who increase their rating over time to be better than predicted, as well as respondents who may begin better than expected but, over time, increase to a rating that is even more positive than expected. Negative RS is deemed present when respondents rate their health better than (or closer to) expected early on, but whose ratings decrease over time compared to expected.

To answer the question as to whether response shift depends on perceived health, we modeled the trajectories of RS conditional on the trajectories of EVGGFP using dual trajectory analysis [34].

To answer the question as to who is likely to experience a RS, we created two logistic regression models one to identify predictors of positive RS and one to predict negative RS. Although trajectory analysis is a probabilistic method, in that respondents receive a posterior probability of being in each trajectory group, respondents can be assigned deterministically to a specific group based on their highest probability. Odds ratios and 95% confidence intervals (CI) were estimated for each variable measured at baseline. With five levels and a reasonably normal distribution, EVGGFP can be modeled as continuous with little repercussion [14,15,35].

### Ethical considerations

Ethics approval for the Cohort Study was obtained from the University of Manitoba Health Research Ethics Board.

## Results

### Description of the cohort

Table 1 gives the characteristics of the sample; the mean age was 40 years, 60% were women, and there were similar numbers of respondents with Crohn’s disease and ulcerative colitis. Over half of the sample was working full time at baseline but 33% of respondents had missed work in the previous 6 months because of IBD.

**Table 1:** **Characteristics of IBD cohort study participants (n = 388)**

**Characteristic**	**N missing**	**N or Mean (SD)**	**Proportion**
Sex: (men/women)	0	157/231	40%/60%
Age at baseline: Mean (SD)	5	40.4 (14.5)	
<25/25–64/≥65	5	57/305/21	15%/80%/5%
Age at diagnosis: Mean (SD)	9	36.0 (14.6)	
Diagnosis	0		
Crohn’s		187	48%
Ulcerative colitis [1]		187	48%
Indeterminate IBD		14	4%
Education: High School or less/Trade School or Diploma/University	35	137/119/97	39%/34%/27%
Employment:	6		
Working full time		204	53%
In school		95	25%
Working part-time		34	9%
Homemaker		26	7%
Retired		20	5%
Disabled		3	<1%
Worked in last year	7	313	82%
Missed work in last 6 months due to IBD	37	116	33%

[1] 18 of the 187 are indeterminate collitis.

### Ratings of health

Table 2 presents the proportion of respondents who were concordant on rating of health (EVGGFP) at study entry and 2 years later. Half reported the same level of health at study entry and at two years follow-up, while 27% reported better health, and 23% deterioration. Of the 90 respondents who rated their health as Very Good at study entry, 10 (11.1%) rated their health higher at the 2-year follow-up, while 32 (35.6%) rated their health lower.

**Table 2:** **SF-36 General health question (EVGGFP) at study entry and 2 years later among 289 (74**%**) with ratings at both measurement occasions**

**Rating at Cohort entry (n)**	**Rating at 2 years**		
	**Concordant (n = 144, 49.8%** **)**	**Increased (n = 78, 27.0%** **)**	**Decreased (n = 67, 23.2%** **)**
Excellent (12)	4 (33.3%)	--	8 (66.7%)
Very good (90)	48 (53.3%)	10 (11.1%)	32 (35.6%)
Good (116)	64 (55.2%)	28 (24.1%)	24 (20.7%)
Fair (52)	24 (46.2%)	25 (48.1%)	3 (5.8%)
Poor (19)	4 (21.1%)	15 (78.9%)	--

Proportions are row percents among persons at each of the five levels of outcome.

### Time-dependent measures

Table 3 presents the distribution of those variables measured at each assessment: symptoms, functioning, health perception, and social support. For the SF-36 subscales, Canadian population norms are presented. For most of the subscales, the IBD cohort members had considerably lower values.

**Table 3:** **Disease activity, symptoms, functioning and social support at study entry and at one- and two-year follow-ups**

**Predictor [norm when available]**	**Study entry**		**Year 1 follow-up**		**Year 2 follow-up**	
	**n**	**Mean (SD)**	**n**	**Mean (SD)**	**n**	**Mean (SD)**
*SF-36 subscales: scored 0–100, higher scores correspond to better HRQL*						
General health perception [77]	284	54.9 (21.3)	357	53.0 (21.4)	342	56.9 (22.3)
Physical function [86]	335	81.0 (21.5)	354	82.5 (21.8)	345	83.2 (22.0)
Role physical [82]	379	69.1 (29.0)	357	75.2 (26.7)	346	77.1 (25.1)
Bodily pain [76]	351	58.3 (23.8)	360	61.8 (21.7)	348	62.7 (22.1)
Vitality [66]	380	49.5 (20.8)	359	51.8 (21.8)	345	51.5 (22.8)
Social function [86]	381	67.4 (27.9)	358	79.5 (24.8)	347	79.2 (23.3)
Role emotional [84]	379	78.7 (23.1)	360	83.0 (22.2)	347	83.3 (21.2)
Mental health [78]	379	67.6 (17.3)	360	71.5 (17.3)	345	71.7 (18.4)
*IBDQ subscales: scored 0 to 7, higher scores correspond to better HRQL*						
Bowel symptoms	364	5.3 (1.1)	350	5.4 (1.1)	343	5.5 (1.1)
Emotional health	338	5.2 (1.1)	354	5.4 (1.1)	344	5.5 (1.0)
Social function	346	5.9 (1.3)	336	6.1 (1.3)	336	6.2 (1.1)
Systemic symptoms	365	4.4 (1.3)	352	4.6 (1.3)	344	4.7 (1.3)
*Brief symptom inventory: scaled 0 to 100, higher scores indicate worse symptoms*						
Global	381	13.7 (12.0)	358	12.9 (12.1)	351	12.2 (10.8)
Somatization	379	17.2 (15.0)	358	16.2 (15.6)	351	14.8 (15.1)
Somatization excluding nausea	379	15.9 (14.9)	358	14.5 (15.2)	351	13.6 (14.9)
Obsessive compulsive	381	21.2 (18.1)	358	21.2 (18.9)	351	20.0 (16.4)
Sensitivity	381	14.7 (17.6)	358	13.4 (16.8)	352	13.1 (16.0)
Depression	381	15.6 (18.3)	358	12.5 (16.5)	352	12.9 (15.0)
Anxiety	380	13.3 (14.6)	358	12.8 (14.8)	352	11.3 (13.0)
Hostility	381	13.0 (13.9)	358	11.8 (12.5)	351	11.1 (11.8)
Phobic	379	5.1 (10.6)	358	5.1 (12.1)	351	4.0 (9.0)
Paranoid	380	9.4 (12.8)	358	9.6 (13.8)	352	9.9 (13.9)
Psychoticism	381	7.0 (11.2)	358	7.1 (11.0)	351	7.6 (11.3)
*MSPSS social support: higher scores correspond to greater support. Total is scored 12–84*						
Total	341	66.2 (14.1	358	65.4 (16.1)	349	65.7 (16.5)
Significant other (1–7)	346	5.8 (1.5)	358	5.8 (1.6)	349	5.8 (1.6)
Family (1–7)	374	5.6 (1.4)	359	5.5 (1.5)	351	5.5 (1.5)
Friends (1–7)	375	5.2 (1.5)	359	5.1 (1.5)	351	5.1 (1.6)
*CPSS Stress scale: scored 0–56, higher scores correspond to greater perceived stress*						
CPSS total	371	22.4 (8.2)	356	21.4 (8.5)	344	20.9 (8.7)

### Predictive model of health

The model used to predict health perception is presented in Table 4. Of the 388 respondents in the cohort, three had no measurement occasions with the EVGGFP question answered and were removed from further analysis, as were three assessments where data were missing. The final model was based on 1691 records among 385 respondents. Most respondents (87% of the 388) had the EVGGFP outcome available for at least four measurement occasions. The predictive model explained 51.3% of the variation in EVGGFP (AIC = 3123.8, BIC = 3206.8 for model with predictors compared to AIC = 3883.1, BIC = 3894.9 for the null model). Symptom frequency and duration (both over the past 6 months and over the past 2 weeks) were significant predictors of EVGGFP and had initially been included, but as their impacts changed over time (and as such are part of the RS) these predictors were dropped from the model and others, including a binary indicator of active disease, were allowed to enter.

**Table 4:** **Best predictive model of EVGGFP over time**

**Variable**	**Scale**	**Beta (SE)**	**p**
Active disease	Y/N	0.242 (0.039)	<.0001
IBDQ systemic	0–6	0.070 (0.022)	0.0014
SF-36 vitality	100–0	0.010 (0.001)	<.0001
SF-36 pain	100–0	0.006 (0.001)	<.0001
BSI somatization*	0–83.33	0.005 (0.002)	0.0021
BSI psychoticism	(30–85) vs (0–25)	0.207 (0.070)	0.0030
SF-36 physical function	100–0	0.008 (0.001)	<.0001
IBDQ social function	6.5 v 7	0.047 (0.043)	0.2702
	5.5–6.5 v 7	0.086 (0.049)	0.0792
	<4.5 v 7	0.133 (0.061)	0.0295

Predictors modeled with lower as the better score and higher as worse. Gender was also included, as it explained variance, but only time-dependent variables contribute to the residuals that are modeled. BSI = Brief Symptom Inventory; IBDQ = Inflammatory Bowel Disease Questionnaire.

*excluding nausea/upset stomach.

### Group-based trajectory model of residuals

**Figure 2:**
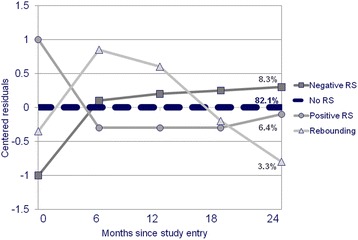
Four-trajectory model of response shift.

The group-based trajectory model was based on 359 of 385 respondents who had at least three residuals. We first considered a 6-trajectory group model which statistically fit the data well (AIC −902.33, BIC (n = 1653 based on records) -991.60, BIC (n = 359 based on persons) -966.40); however, two of the trajectories (representing less than 10% of the sample) showed no consistent pattern and variation was within the precision of the measurement scale (1 unit). Table 5 shows the fit parameters of the final, four-trajectory group model (AIC-963.32, BIC (n = 1653)-1017.42, BIC (n = 359) -1002.15) which also showed very good fit: all mean posterior probabilities were greater than 0.90, and theoretical and assigned proportions were almost identical. Figure 2 shows the four trajectories. Because of the centering of the residuals prior to modeling, a flat line at zero does not necessarily indicate agreement with the predicted score, but rather consistency over time in the difference between the observed and expected scores. The majority of the sample (82%) was part of the flat trajectory at 0, indicating no RS. A small proportion (6%) was part of a trajectory with centered residuals that dropped over the first six months, generally respondents who started with health ratings worse than predicted but that increased with respect to predicted; this group is labeled as showing positive RS. A further 8% started with scores generally better than predicted, but decreased their health rating compared to predicted over the next six months, labeled negative RS. Another 3% started better than expected but rated themselves considerably worse at 6 months, but with a return thereafter; this group is labeled as having a re-bound response-shift pattern.

**Table 5:** **Trajectory groups of RS categories**

	**No RS detected**	**Negative RS**	**Positive RS**	**Rebounded**
Theoretical%	82.1	8.3	6.4	3.3
N (%) based on assignment	294 (81.9%)	31 (8.6%)	23 (6.4%)	11 (3.1%)
Range of centered residuals				
Mean (SD)	0.89 (0.34)	2.00 (0.45)	2.22 (0.59)	2.03 (0.50)
Min-Max	0.07–1.58	1.43–3.11	1.59–3.67	1.35–3.02
Posterior probability of belonging to RS group (>0.7 considered good fit)				
Mean (SD)	0.99 (0.04)	0.92 (0.16)	0.95 (0.11)	0.93 (0.15)

Modelled on 1,653 observations among 359 subjects with at least 3 outcomes at interviews with at least half the predictors.

### Observed versus predicted health

**Figure 3:**
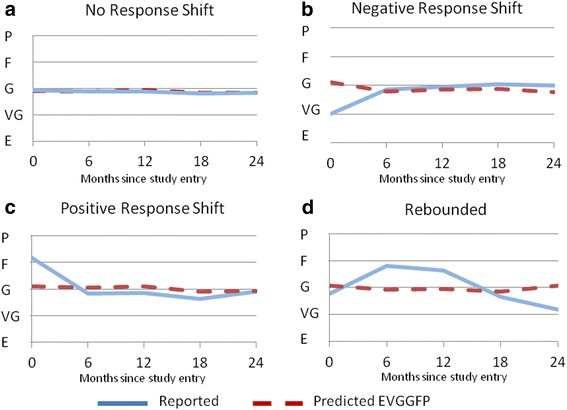
Observed compared to expected health-related quality of life.

A graph of observed and predicted EVGGFP by trajectory group is presented in Figure 3. All trajectory groups follow a similar predicted course over time. The trajectories of observed and predicted health ratings among the 82% classified as not having experienced RS are essentially identical (Figure 3a). However, among the 8% with negative RS, it can be seen that, while health was rated higher at the first interview, ratings reached a plateau similar to predicted by 6 months (Figure 3b). Among the 6% classified as having positive RS, while they began poorly compared to predicted, by 6 months they reached an average rating slightly better than expected (Figure 3c). The rebound group (3%) rated health worse than predicted by 6 months, but higher than predicted at 2 years (Figure 3d).

### Dual trajectory analysis

**Figure 4:**
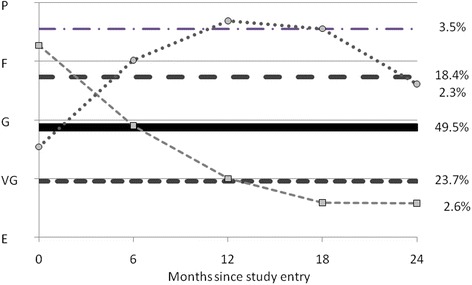
Six-group model of EVGGFP.

Figure 4 shows the trajectories of the health rating over time (EVGGFP). There were four flat groups, 24% at VG, 50% near G, 18% near F and 4% between F and P, and two changing groups, one (3%) with improving health rating and one with predominantly decreasing health rating (2%).

Results from the dual trajectory analysis of EVGGFP modeled conditionally on RS group indicate that only four trajectories of health could be modeled jointly, with some small redistribution of people into trajectories. These are: three flat groups, one between poor and fair (20%) and two others at good (51%) and at VG (24%), and the remaining 5% of the sample with improving health ratings from F to VG over the two year period.

**Figure 5:**
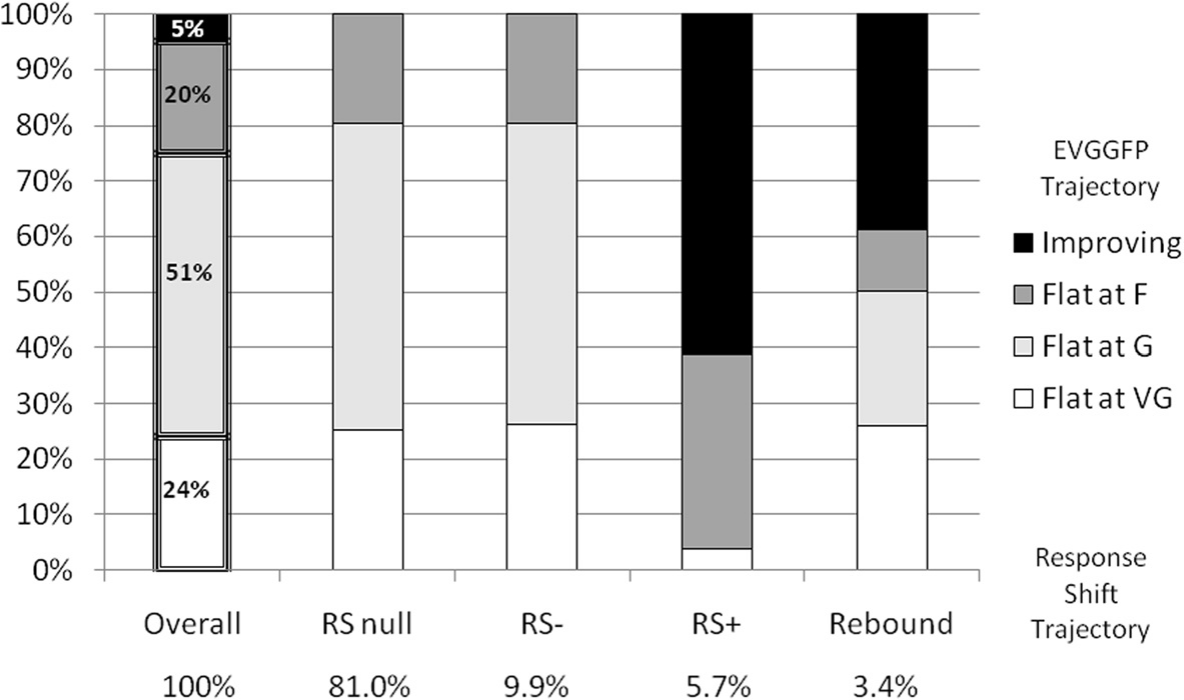
EVGGFP conditional on response shift from dual trajectory model.

Figure 5 shows the distribution of health rating trajectories over RS groups, first over the full sample to serve as an expected distribution without considering RS and then for each RS group separately. Approximately 24% rated their health as stable at VG and this distribution was similar for all RS groups except for the positive RS group. Overall, only a small proportion of respondents (5%) rated their health as improving but 62% of RS positive and 40% of RS rebound did so, recognizing that both these groups are small (6% and 4% of all groups, respectively).

### Predictors of RS

Predictors of RS are presented in Table 6. Univariate comparisons of the 23 respondents assigned to the positive RS group with the 294 in the RS null group indicate that those with positive RS are more likely to be younger (on average, diagnosed at 30 compared to 38, for an odds ratio of 0.96 associated with each additional year of age), to have more hostility symptoms and to report worse scores for pain, mental health, social and role physical functioning and general health perception at baseline. As higher scores on the SF-36 signify better HRQL, the risk of positive RS was lower when baseline scores were higher. Thus, lower baseline scores on the GH subscale were more likely among respondents with positive RS. Worse baseline pain and social function scores were also found to be associated with the presence of negative as well as positive RS. Gender, vitality, physical function, and measures of personality and coping type were not found to have an association with RS.

**Table 6:** **Baseline predictors of response shift**

	**Negative (n = 31) v no RS (n = 294)**		**Positive (n = 23) v no RS (n = 294)**	
	**Odds ratio (95% CI) [per unit of predictor]**	**Mean (RS-) versus mean (RS**_**0**_ **) (pooled SD) p-value of the difference in means**	**Odds ratio (95% CI) [per unit of predictor]**	**Mean (RS-) versus mean (RS**_**0**_ **) (pooled SD) p-value of the difference in means**
Age at diagnosis: older age at diagnosis reduces the risk of RS+	-		0.96 (0.93, <1.00)	30 v 38 (14.8) p = 0.0216
BSI Hostility: more hostility symptoms (score ≥35) at baseline increases the risk of RS+			3.63 (1.11, 11.94)	17% v 5% scored above 35 P = 0.0477^
**SF-36 (OR based on a 10-pt difference)**				
GH:	-			
Higher GH at baseline reduces risk of RS+			0.74	41.6 v 55.5 (21.5) p = 0.0072
			(0.59, 0.93)	
Pain:				
Lower pain at baseline reduces risk for RS- and RS+	0.86	51.1 v 60.3 (2.4)	0.79	45.5 v 60.3 (24.9) p = 0.0080
	(0.74, 1.01)	p = 0.0593	(0.66,0.95)	
MH:	-		|	
Better MH at baseline reduces risk of RS+			0.76 (0.60, 0.95)	59.6 v 68.7 (17.3) p = 0.0152
Social:				
Better social function at baseline reduces the risk of RS- and RS+	0.88	58.9 v 69.3 (27.7) p = 0.0465	0.87	57.6 v 69.3 (27.9) p = 0.0533
	(0.77, 1.00)		(0.75, 1.00)	
RP:	-			
Better role physical at base-line reduces the risk of RS+			0.87 (0.76,<1.00)	56.8 v 70.1 (29.8) p = 0.0398

All comparisons are univariate ^using Fisher’s exact test.

CI: confidence interval.

## Discussion

This study found no evidence of RS in 82% of the cohort on one of the most frequently asked questions in health. This suggests that this is a good question to ask over time in this population.

Positive RS was experienced by 6% of the IBD sample and negative RS by 8% over a two-year period. These RS rates are lower than were found over one year in a sample of incident stroke (14% positive and 15% negative), but higher than were found in a volunteer study of prevalent MS. While the diagnosis of a chronic disease such as IBD would be expected to be a sufficient catalyst to initiate a RS, it is not surprising that stroke, an event with a very precise onset that can produce serious health consequences in the blink of an eye, would be a stronger catalyst, resulting in more RS, even over a shorter time period. Also, while this IBD cohort was established within seven years of diagnosis (mean years since diagnosis = 4, SD = 2), it may be that RS occurs early and was missed in some respondents. Both the positive and negative RS found in this study occurred by 6 months of follow-up. We previously detected almost no RS in a group with prevalent MS [15]. It was impossible to determine in that sample whether that was because no RS had occurred or whether it was just not detected because it had occurred prior to the measurement occasions, or even because of arbitrary measurement timing, chosen by the volunteer.

It is not possible, using this method, to identify which type of RS (reconceptualization, reprioritization, or recalibration) occurred and it would take qualitative work to untangle what drove the residual value. In our previous work [14], we found support for recalibration because of validation with the then-test.

RS was found to be associated with health perception. Several other predictors of RS were identified. Worse baseline pain and social function scores were found to be associated with the presence, but not direction, of RS: RS was more likely among those in either the positive or negative RS groups compared to those without RS. Those identified with positive response shift were also more likely to be younger, as well as having worse mental health, role physical functioning and general health perception scores and more hostility symptoms at baseline. These results are concordant with the finding from Lix et al. using discriminative analysis and logistic regression; this method identified pain and social function as predictors of reprioritization RS [39].

The EVGGFP question was selected as the outcome rather than the general health subscale for reasons of predictors to evaluate RS. While reports of symptoms, functioning, and perceived stress and social support may be good predictors for the rating of heath [20,22,23], they would not be expected to predict some of the other constructs in the GH subscale such as the extent to which people expect their health to worsen.

Foundational work on the meaning of self-rated health [36,37] indicates that people do not use the same frame of reference when answering the global health status item. Most commonly the rating is influenced by physical health including physical function and general physical condition; presence of positive health behaviours and absence of negative behaviours also play role as do health comparisons with social group. This early research has also shown that the frame of reference used did not depend on the person's rating of their health. In longitudinal studies, it would be relevant to ask whether the person’s frame of reference changes over time, however, change in frame of reference is a mechanism of response shift [38]. Further research on the propensity for people to make a response shift is warranted.

A limitation of this analysis is that all predictors were obtained by self-report and could themselves have been influenced by RS. Although measured variables were included as part of the Cohort Study data collection, none were observed at all measurement occasions. While fixed predictors may increase the variation in the outcome explained by the predictive model, they do not contribute to the determination of RS as they do not contribute to discrepancies over time between observed and predicted scores.

This is not the first study to identify the presence of RS in individuals with IBD. Previous research to develop a new method of detecting reprioritization RS [39] also used data from the IBD Cohort Study, at the baseline and six month measurement occasions. RS was identified in some of the SF-36 subscales.

These results, combined with the current ones, suggest that some individuals with chronic conditions that are associated with exacerbations and remissions of symptoms over time are susceptible to RS. These flares in activity may serve as catalysts for RS [38]. However, even individuals not currently experiencing disease exacerbations have been shown to be susceptible to RS [40], perhaps because their expectations for high symptom burden were not realized.

In conclusion, the majority of people with IBD did not demonstrate a RS indicating that the health rating over time was stable in relation to that predicted by known time varying clinical variables. This adds to the evidence that the single question on self-rated health is useful for monitoring individuals over time [41].
